# Checklist of the family Sciaridae (Diptera) of Finland

**DOI:** 10.3897/zookeys.441.7381

**Published:** 2014-09-19

**Authors:** Pekka Vilkamaa

**Affiliations:** 1 Finnish Museum of Natural History, Zoology Unit, P.O. Box 17, FI-00014 University of Helsinki, Finland

**Keywords:** Checklist, Finland, Diptera, Sciaridae, *Sciarosoma*

## Abstract

A checklist of the family Sciaridae (Diptera) recorded from Finland is provided. The genus *Sciarosoma* Chandler with a disputed family placement is also included in the list.

## Introduction

Sciaridae, the black fungus gnats, is one of the large families in Sciaroidea, little studied and notoriously difficult in its taxonomy. Up-to-date keys are not available for all European species, but various publications must be consulted for identification. Our knowledge of Finnish fauna stems from two classic publications: Frey (1948) and Tuomikoski (1960) but Menzel and Mohrig (2000) made fundamental nomenclatorial changes in their study of the type materials of all Palaearctic species.

As no records of Sciaridae in Finland were published between the publication of Tuomikoski’s (1960) monograph on the family and Hackman’s (1980) check-list of Finnish nematoceran Diptera, the species lists in both works are essentially unchanged, despite some obvious lapses in the latter. Our knowledge of Finnish fauna has since grown mainly in the taxonomic treatment of various genera and species groups (see Vilkamaa et al. 2013). All species recorded in Finland in publications thus far, besides the most recent ones, appear in H. Silfverberg’s compilations of changes to the Finnish insect fauna published every five years (for the latest one, see Silfverberg 2012).

With regard to nomenclature and generic classification, the present list follows the revisions of Menzel and Mohrig (2000) and Mohrig et al. (2013), although on the basis of phylogenetic analyses *Baeosciara* Tuomikoski, *Dolichosciara* Tuomikoski, *Peyerimhoffia* Kieffer, *Prosciara* Frey and *Mouffetina* Frey are regarded as genera (see Vilkamaa 2000, 2003, Vilkamaa and Hippa 2005, Shin et al. 2013). For names currently understood as junior synonyms, mostly only the names listed as valid in Tuomikoski (1960) are mentioned. For complete synonym lists, one can consult Menzel and Mohrig (2000) and Mohrig et al. (2013). Although some genera can be regarded as relatively well-known for the Finnish species they contain, for some others, such as the species-rich *Bradysia* Winnertz, many new species including undescribed ones may be present in the native fauna. Even so, together with Germany, Sweden and the United Kingdom, Finland can be considered one of the countries best known for its sciarid fauna.

**Number of species:**

World: 2600 species

Europe: 700 species

Finland: 338 species

Faunistic knowledge level in Finland: average

## Checklist

Nematocera Dumeril, 1805

infraorder Bibionomorpha Hennig, 1954

superfamily Sciaroidea Billberg, 1820

**SCIARIDAE** Billberg, 1820

***BAEOSCIARA*** Tuomikoski, 1960

*Baeosciara discolor* (Lengersdorf, 1928)

= *pusillima* (Frey, 1948)

*Baeosciara sinuata* (Menzel & Mohrig, 1997)

*Baeosciara scotica* (Edwards, 1925)

***BRADYSIA*** Winnertz, 1867

*Bradysia affinis* (Zetterstedt, 1838)

= *pratincola* Tuomikoski, 1960

*Bradysia albanensis* (Lengersdorf, 1926)

*Bradysia alpicola* (Winnertz, 1867)

= *mutabilis* (Lengersdorf, 1926)

= *morio* auct. nec (Fabricius, 1794)

*Bradysia angustata* Tuomikoski, 1960

*Bradysia angustostylata* Menzel, 2005

*Bradysia aprica* (Winnertz, 1867)

*Bradysia arcana* Menzel & Mohrig, 1998

= *fenestralis* auct. nec (Zetterstedt, 1838)

*Bradysia arcula* Vilkamaa, Salmela & Hippa, 2007

*Bradysia ascenda* Rudzinski, 1994

*Bradysia bicolor* (Meigen, 1818)

*Bradysia bispinifera* Mohrig & Krivosheina, 1983

*Bradysia brevispina* Tuomikoski, 1960

*Bradysia browni* (Shaw, 1935)

= *diversiabdominalis* (Lengersdorf, 1941)

= *laurencei* Menzel & Mohrig, 2000

*Bradysia cinerascens* (Grzegorzek, 1884)

= *lanicauda* Tuomikoski, 1960

*Bradysia confinis* (Winnertz, 1867)

*Bradysia excelsa* Menzel & Mohrig, 1998

*Bradysia flavipila* Tuomikoski, 1960

*Bradysia forcipulata* (Lundbeck, 1898)

*Bradysia forficulata* (Bezzi, 1914)

= *nocturna* Tuomikoski, 1960

*Bradysia fungicola* (Winnertz, 1867)

*Bradysia giraudii* (Egger, 1862)

*Bradysia globulifera* (Lengersdorf, 1934)

*Bradysia hilariformis* Tuomikoski, 1960

*Bradysia hilaris* (Winnertz, 1867)

*Bradysia holsatica* Heller, 2004

*Bradysia hortensis* Heller, 2000

*Bradysia impatiens* (Johannsen, 1912)

= *difformis* (Frey, 1948)

= *paupera* Tuomikoski, 1960

*Bradysia inusitata* Tuomikoski, 1960

*Bradysia iridipennis* (Zetterstedt, 1838)

*Bradysia lapponica* (Lengersdorf, 1926)

*Bradysia latiterga* Tuomikoski, 1960

*Bradysia leptoptera* Tuomikoski, 1960

*Bradysia lilienthalae* Mohrig & Menzel, 1990

*Bradysia lobulifera* Frey, 1948

*Bradysia longicauda* Mohrig & Menzel, 1990

*Bradysia longicubitalis* (Lengersdorf, 1924)

= *cinereovittata* Frey, 1948

*Bradysia minima* Mohrig & Mamaev, 1989

*Bradysia moesta* Frey, 1948

= *albosetosa* Frey, 1948

*Bradysia moestula* Tuomikoski, 1960

*Bradysia nervosa* (Meigen, 1818)

*Bradysia nitidicollis* (Meigen, 1818)

= *atroparva* Frey, 1948

*Bradysia normalis* Frey, 1948

*Bradysia ocellaris* (Comstock, 1882)

*Bradysia pallipes* (Fabricius, 1787)

= *brunnipes* (Meigen, 1804)

= *picipes* (Zetterstedt, 1838)

*Bradysia pauperata* (Winnertz, 1867)

*Bradysia peraffinis* Tuomikoski, 1960

*Bradysia pilistriata* Frey, 1948

*Bradysia placida* (Winnertz, 1867)

= *fimbricauda* Tuomikoski, 1960

*Bradysia polonica* (Lengersdorf, 1929)

*Bradysia praecox* (Meigen, 1818)

*Bradysia rectinervis* Frey, 1948

*Bradysia reflexa* Tuomikoski, 1960

*Bradysia regularis* (Lengersdorf, 1934)

= *subnervosa* Frey, 1948

*Bradysia rufescens* (Zetterstedt, 1852)

*Bradysia scabricornis* Tuomikoski, 1960

= *subscabricornis* Mohrig & Menzel, 1990

*Bradysia siberica* Komarova, 2001

*Bradysia spinostyla* Mohrig & Menzel, 1990

*Bradysia strigata* (Staeger, 1840)

*Bradysia subalpina* Frey, 1948

*Bradysia subamoena* Mohrig & Krivosheina, 1989

*Bradysia submoesta* Mohrig & Krivosheina, 1989

*Bradysia tilicola* (Loew, 1850)

= *amoena* (Winnertz, 1867)

= *cellarum* Frey, 1948

*Bradysia trivittata* (Staeger, 1840)

*Bradysia vagans* (Winnertz, 1868)

= *callicera* Frey, 1948

*Bradysia vernalis* (Zetterstedt, 1851)

*Bradysia zonata* Rudzinski, 1993

***BRADYSIOPSIS*** Tuomikoski, 1960

*Bradysiopsis vittata* (Meigen, 1830)

= *leucotricha* (Tuomikoski, 1960)

*Bradysiopsis vittigera* (Zetterstedt, 1851)

***CAMPTOCHAETA*** Hippa & Vilkamaa, 1994

*Camptochaeta austriaca* Heller, 2012

*Camptochaeta bournei* (Shaw, 1941)

*Camptochaeta camptochaeta* (Tuomikoski, 1960)

*Camptochaeta consimilis* (Holmgren, 1869)

*Camptochaeta delicata* (Lengersdorf, 1935)

*Camptochaeta duplicata* Hippa & Vilkamaa, 1994

*Camptochaeta fallax* Hippa & Vilkamaa, 1994

*Camptochaeta hirtula* (Lengersdorf, 1934)

= *fulvicollis* (Tuomikoski, 1960)

*Camptochaeta propria* Hippa & Vilkamaa, 1994

*Camptochaeta scanica* Hippa & Vilkamaa, 1994

*Camptochaeta sicilicula* Hippa & Vilkamaa, 1994

*Camptochaeta simulator* Hippa & Vilkamaa, 1994

*Camptochaeta stammeri* (Lengersdorf, 1940)

*Camptochaeta tenuipalpalis* (Mohrig & Antonova, 1978)

*Camptochaeta uniformis* (Mohrig & Menzel, 1990)

*Camptochaeta vivax* (Frey, 1948)

*Camptochaeta xystica* Hippa & Vilkamaa, 1994

***CHAETOSCIARA*** Frey, 1942

*Chaetosciara estlandica* (Lengersdorf, 1929)

***CLAUSTROPYGA*** Hippa, Vilkamaa & Mohrig, 2003

*Claustropyga acanthostyla* (Tuomikoski, 1960)

*Claustropyga brevichaeta* (Mohrig & Antonova, 1978)

*Claustropyga clausa* (Tuomikoski, 1960)

*Claustropyga corticis* (Mohrig & Antonova, 1978)

*Claustropyga ctenophora* Hippa, Vilkamaa & Mohrig, 2003

*Claustropyga heteroclausa* (Rudzinski, 1991)

*Claustropyga refrigerata* (Lengersdorf, 1930)

*Claustropyga subcorticis* (Mohrig & Krivosheina, 1985)

***CORYNOPTERA*** Winnertz, 1867

*Corynoptera barbata* Tuomikoski, 1960

*Corynoptera bicuspidata* (Lengersdorf, 1926)

= *gymnops* Tuomikoski, 1960

*Corynoptera bipartita* Mohrig & Krivosheina, 1985

*Corynoptera blanda* (Winnertz, 1867)

*Corynoptera boletiphaga* (Lengersdorf, 1940)

= *geogenia* Tuomikoski, 1960

*Corynoptera breviformis* Mohrig & Krivosheina, 1983

*Corynoptera cracentis* Vilkamaa, Hippa & Heller, 2013

*Corynoptera cuniculata* (Lengersdorf, 1942)

= *caldariorum* Tuomikoski, 1960

*Corynoptera defecta* (Frey, 1948)

*Corynoptera dentata* (Bukowski & Lengersdorf, 1936)

*Corynoptera deserta* Heller & Menzel, 2006

= *minutula* (Bukowski & Lengersdorf, 1936)

*Corynoptera dubitata* Tuomikoski, 1960

*Corynoptera fera* Mohrig & Heller, 1992

*Corynoptera flavicauda* (Zetterstedt, 1855)

*Corynoptera forcipata* (Winnertz, 1867)

*Corynoptera furcifera* Mohrig & Mamaev, 1987

*Corynoptera globiformis* (Frey, 1945)

*Corynoptera hypopygialis* (Lengersdorf, 1926)

= *piniphila* (Lengersdorf, 1940)

*Corynoptera inari* Vilkamaa, Hippa & Heller, 2013

*Corynoptera inexspectata* Tuomikoski, 1960

*Corynoptera irmgardis* (Lengersdorf, 1930)

*Corynoptera levis* Tuomikoski, 1960

*Corynoptera luteofusca* (Bukowski & Lengersdorf, 1936)

*Corynoptera marinae* Mohrig & Krivosheina, 1986

*Corynoptera melanochaeta* Mohrig & Menzel, 1992

*Corynoptera membranigera* (Kieffer, 1903)

= *trispina* Tuomikoski, 1960

*Corynoptera montana* (Winnertz, 1869)

*Corynoptera ninae* Hippa, Vilkamaa & Heller, 2010

*Corynoptera obscuripila* Tuomikoski, 1960

*Corynoptera parvula* (Winnertz, 1867)

= *uncinata* (Hippa & Vilkamaa, 1994)

*Corynoptera parvulaformis* Mohrig, 1985

*Corynoptera penna* (Pettey, 1918)

= *alneti* Hippa, Vilkamaa & Heller, 2010

*Corynoptera perochaeta* (Mohrig & Menzel, 1990)

*Corynoptera plusiochaeta* Hippa, Vilkamaa & Heller, 2010

*Corynoptera polana* Rudzinski, 2009

*Corynoptera postforcipata* Rudzinski, 1993

*Corynoptera postglobiformis* Mohrig, 1993

*Corynoptera praeforcipata* Mohrig & Mamaev, 1987

*Corynoptera quantula* (Hippa & Vilkamaa, 1994)

*Corynoptera saccata* Tuomikoski, 1960

*Corynoptera saetistyla* Mohrig & Krivosheina, 1985

*Corynoptera salmelai* Vilkamaa, Hippa & Heller, 2013

*Corynoptera sphenoptera* Tuomikoski, 1960

*Corynoptera spiciforceps* Vilkamaa, Hippa & Heller, 2013

*Corynoptera spinifera* Tuomikoski, 1960

*Corynoptera subblanda* Tuomikoski, 1960

*Corynoptera subdentata* Mohrig, 1985

*Corynoptera subparvula* Tuomikoski, 1960

*Corynoptera subsedula* Mohrig & Mamaev, 1987

*Corynoptera subtetrachaeta* Komarova, 1995

*Corynoptera subtilis* (Lengersdorf, 1929)

= *longicornis* (Bukowski & Lengersdorf, 1936)

*Corynoptera subvariegata* Rudzinski, 1992

*Corynoptera tetrachaeta* Tuomikoski, 1960

*Corynoptera trepida* (Winnertz, 1867)

= *clinochaeta* Tuomikoski, 1960

*Corynoptera triacantha* Tuomikoski, 1960

*Corynoptera tumidula* Hippa, Vilkamaa & Heller, 2010

*Corynoptera tuomikoskii* Hippa, Vilkamaa & Heller, 2013

*Corynoptera unidentata* (Hippa & Vilkamaa, 1994)

*Corynoptera vagula* Tuomikoski, 1960

*Corynoptera verrucifera* (Lengersdorf, 1952)

*Corynoptera voluptuosa* Mohrig & Mamaev, 1987

*Corynoptera waltraudis* Mohrig & Mamaev, 1987

*Corynoptera winnertzi* Mohrig, 1993

***COSMOSCIARA*** Frey, 1942

*Cosmosciara perniciosa* (Edwards, 1922)

***CRATYNA*** Winnertz, 1867

= *Plastosciara* Berg, 1899

= *Decembrina* Frey, 1948

= *Dendrosciara* Frey, 1948

**sg. *Cratyna*** Winnertz, 1867

*Cratyna ambigua* (Lengersdorf, 1934)

= *latiforceps* (Bukowski & Lengersdorf, 1936)

*Cratyna atra* Winnertz, 1867

= *pictiventris* (Kieffer, 1898)

*Cratyna betulae* (Mohrig & Mamaev, 1992)

*Cratyna breviflagellata* (Mohrig & Mamaev, 1985)

*Cratyna monumenta* Rudzinski, 2009

*Cratyna pernitida* (Edwards, 1915)

*Cratyna schineri* (Winnertz, 1867)

*Cratyna sicata* Vilkamaa, Hippa & Heller, 2013

*Cratyna symplecta* (Rudzinski, 1991)

*Cratyna uliginosa* (Lengersdorf, 1929)

*Cratyna vaporariorum* (Frey, 1948)

**sg. *Diversicratyna*** Menzel & Mohrig, 1998

*Cratyna spiculosa* (Rudzinski, 1993)

**sg. *Spathobdella*** Frey, 1948

*Cratyna colei* (Freeman, 1990)

= *brachialis* auct. nec (Winnertz, 1871)

*Cratyna falcata* (Tuomikoski, 1960)

*Cratyna falcifera* (Lengersdorf, 1933)

*Cratyna longispina* (Pettey, 1918)

= *tuberculata* (Tuomikoski, 1960)

*Cratyna nobilis* (Winnertz, 1867)

= *brachialis* (Winnertz, 1867)

*Cratyna perplexa* (Winnertz, 1867)

= *socialis* (Winnertz, 1871)

= *brevicornis* (Tuomikoski, 1957)

***CTENOSCIARA*** Tuomikoski, 1960

*Ctenosciara exigua* Salmela & Vilkamaa, 2005

*Ctenosciara hyalipennis* (Meigen, 1804)

***DICHOPYGINA*** Vilkamaa, Hippa & Komarova, 2004

*Dichopygina aculeata* Vilkamaa, Hippa & Komarova, 2004

*Dichopygina intermedia* (Mohrig & Krivosheina, 1982)

*Dichopygina nigrohalteralis* (Frey, 1948)

*Dichopygina ramosa* Vilkamaa, Hippa & Komarova, 2004

***DOLICHOSCIARA*** Tuomikoski, 1960

*Dolichosciara flavipes* (Meigen, 1804) 185

*Dolichosciara hippai* Komarova & Vilkamaa, 2006

*Dolichosciara nigrovittata* (Strobl, 1910)

*Dolichosciara orcina* (Tuomikoski, 1960)

*Dolichosciara ornata* (Winnertz, 1867)

*Dolichosciara saetosa* (Lengersdorf, 1929)

*Dolichosciara spissispina* Vilkamaa, Hippa & Heller, 2013

***EPIDAPUS*** Haliday, 1851

**sg. *Epidapus*** Haliday, 1851

*Epidapus alnicola* (Tuomikoski, 1957)

*Epidapus atomarius* (De Geer, 1778)

*Epidapus gracilis* (Walker, 1848)

*Epidapus ignotus* (Lengersdorf, 1942)

= *gracilior* (Tuomikoski, 1960)

*Epidapus microthorax* (Börner, 1903)

= *gracilicornis* (Lengersdorf, 1926)

*Epidapus schillei* (Börner, 1903)

= *titan* Frey, 1948

= *intermittens* Tuomikoski, 1959

**sg. *Pseudoaptanogyna*** Vimmer, 1926

*Epidapus abieticola* Frey, 1948

*Epidapus bispinulosus* Mohrig & Kauschke, 1994

*Epidapus echinatum* Mohrig & Kozánek, 1992

*Epidapus ignavus* (Lengersdorf, 1941)

***KEILBACHIA*** Mohrig, 1987

*Keilbachia ferrata* (Hippa & Vilkamaa, 1994)

***LEPTOSCIARELLA*** Tuomikoski, 1960

**sg. *Hirtipennia*** Mohrig & Menzel, 1997

*Leptosciarella hirtipennis* (Zetterstedt, 1838)

*Leptosciarella holotricha* Mohrig & Menzel, 1997

**sg. *Leptosciarella*** Tuomikoski, 1960

*Leptosciarella brevior* (Tuomikoski, 1960)

*Leptosciarella brevipalpa* (Mohrig & Menzel, 1992)

*Leptosciarella claviforceps* (Tuomikoski, 1960)

*Leptosciarella dimera* (Tuomikoski, 1960)

*Leptosciarella fuscipalpa* (Mohrig & Mamaev, 1979)

*Leptosciarella helvetica* (Rudzinski, 1992)

*Leptosciarella hispida* (Winnertz, 1871)

*Leptosciarella ignis* Heller, 2012

*Leptosciarella krille* Heller, 2012

*Leptosciarella melanoma* (Mohrig & Menzel, 1990)

*Leptosciarella nudinervis* (Tuomikoski, 1960)

*Leptosciarella pilosa* (Staeger, 1840)

*Leptosciarella reducta* Heller & Menzel, 2013

*Leptosciarella rejecta* (Winnertz, 1867)

*Leptosciarella scutellata* (Staeger, 1840)

= *elegans* (Winnertz, 1867)

*Leptosciarella subcoarctata* Mohrig & Menzel, 1997

*Leptosciarella subpilosa* (Edwards, 1925)

*Leptosciarella subspinulosa* (Edwards, 1925)

*Leptosciarella subviatica* Mohrig & Menzel, 1997

*Leptosciarella trochanterata* (Zetterstedt, 1851)

= *coarctata* (Winnertz, 1867)

*Leptosciarella truncata* (Tuomikoski, 1960)

*Leptosciarella viatica* (Winnertz, 1867)

*Leptosciarella viaticella* Mohrig & Krivosheina, 1979

*Leptosciarella yerburyi* (Freeman, 1983)

**sg. *Leptospina*** Mohrig & Menzel, 1997

*Leptosciarella atricha* (Tuomikoski, 1960)

**sg. *Trichosiopsis*** Tuomikoski, 1960

*Leptosciarella tuberculigera* (Tuomikoski, 1960)

***LYCORIELLA*** Frey, 1942

**sg. *Coelostylina*** Tuomikoski, 1960

*Lycoriella eflagellata* Tuomikoski, 1960

*Lycoriella freyi* Tuomikoski, 1960

**sg. *Hemineurina*** Tuomikoski, 1960

*Lycoriella algida* (Frey, 1948)

*Lycoriella cochleata* (Rübsaamen, 1898)

*Lycoriella conspicua* (Winnertz, 1867)

*Lycoriella inflata* (Winnertz, 1867)

= *venosa* auct. nec (Staeger, 1840)

*Lycoriella modesta* (Staeger, 1840)

*Lycoriella piristylata* Vilkamaa, Hippa & Heller, 2013

*Lycoriella thuringiensis* Menzel & Mohrig, 1991

*Lycoriella vitticollis* (Holmgren, 1883)

= *permutata* (Lundbeck, 1900)

**sg. *Lycoriella*** Frey, 1942

*Lycoriella aberrans* Tuomikoski, 1960

*Lycoriella acutostylia* Mohrig & Menzel, 1990

*Lycoriella agraria* (Felt, 1897)

= *cellaris* (Lengersdorf, 1934)

*Lycoriella approximatonervis* (Frey, 1948)

*Lycoriella brevipila* Tuomikoski, 1960

*Lycoriella inconspicua* Tuomikoski, 1960

*Lycoriella ingenua* (Dufour, 1839)

= *solani* (Winnertz, 1871)

*Lycoriella latilobata* Menzel & Mohrig, 2000

*Lycoriella lundstromi* (Frey, 1948)

*Lycoriella micria* Mohrig & Menzel, 1990

*Lycoriella minutula* Mohrig & Krivosheina, 1987

*Lycoriella pallidior* Tuomikoski, 1960

*Lycoriella parva* (Holmgren, 1869)

= *obscuratipes* (Frey, 1948)

= *curvispina* Tuomikoski, 1960

*Lycoriella sativae* (Johannsen, 1912)

= *castanescens* (Lengersdorf, 1940)

= *fucorum* (Frey, 1948)

*Lycoriella subterranea* (Märkel, 1844)

= *vanderwieli* (Schmitz, 1920)

*Lycoriella tenera* Vilkamaa, Hippa & Heller, 2013

*Lycoriella weberi* Menzel & Heller, 2013

***MOUFFETINA*** Frey, 1942

*Mouffetina expolita* (Coquillett, 1900)

*Mouffetina pulchricornis* (Edwards, 1925)

*Mouffetina silvestris* (Mohrig & Antonova, 1978)

***PEYERIMHOFFIA*** Kieffer, 1903

*Peyerimhoffia crassistylata* (Frey, 1948)

*Peyerimhoffia infera* Vilkamaa & Hippa, 2005

*Peyerimhoffia menzeli* Vilkamaa & Hippa, 2005

*Peyerimhoffia quadrifera* Vilkamaa, Hippa & Heller, 2013

*Peyerimhoffia sepei* Hippa & Vilkamaa, 2005

*Peyerimhoffia thula* Vilkamaa & Hippa, 2005

*Peyerimhoffia vagabunda* (Winnertz, 1867)

= *brachyptera* Kieffer, 1903

***PHYTOSCIARA*** Frey, 1942

*Phytosciara halterata* (Lengersdorf, 1926)

*Phytosciara macrotricha* (Lengersdorf, 1926)

***PNYXIA*** Johannsen, 1912

*Pnyxia scabiei* (Hopkins, 1895)

***PNYXIOPSIS*** Tuomikoski, 1960

*Pnyxiopsis aliger* Tuomikoski, 1960

*Pnyxiopsis degener* (Tuomikoski, 1957)

***PROSCIARA*** Frey, 1942

*Prosciara furtiva* Vilkamaa & Hippa, 1996

*Prosciara plusiochaeta* Hippa & Vilkamaa, 1991

*Prosciara porrecta* (Lengersdorf, 1929)

*Prosciara producta* (Tuomikoski, 1960)

*Prosciara prosciaroides* (Tuomikoski, 1960)

*Prosciara ungulata* (Winnertz, 1867)

***PSEUDOLYCORIELLA*** Menzel & Mohrig, 1998

*Pseudolycoriella brunnea* (Bukowski & Lengersdorf, 1936)

*Pseudolycoriella japonensis* (Mohrig & Menzel, 1992)

*Pseudolycoriella koreensis* (Mohrig & Menzel, 1992)

*Pseudolycoriella monticula* (Mohrig & Menzel, 1992)

*Pseudolycoriella nodulosa* (Mohrig & Krivosheina, 1985)

*Pseudolycoriella paludum* (Frey, 1948)

*Pseudolycoriella subbruckii* (Mohrig & Hövemeyer, 1992)

***SCATOPSCIARA*** Edwards, 1927

**sg. *Scatopsciara*** Edwards, 1927

*Scatopsciara atomaria* (Zetterstedt, 1851)

= *vivida* (Winnertz, 1867)

*Scatopsciara bucera* Rudzinski, 1994

*Scatopsciara calamophila* Frey, 1948

*Scatopsciara curviforceps* (Bukowski & Lengersdorf, 1936)

= *myrmecophila* Frey, 1948

*Scatopsciara edwardsi* Freeman, 1983

*Scatopsciara fluviatilis* (Lengersdorf, 1940)

*Scatopsciara geophila* (Tuomikoski, 1960)

*Scatopsciara multispina* (Bukowski & Lengersdorf, 1936)

*Scatopsciara neglecta* Menzel & Mohrig, 1998

*Scatopsciara pusilla* (Meigen, 1818)

*Scatopsciara subcalamophila* Menzel & Mohrig, 1991

*Scatopsciara subciliata* Tuomikoski, 1960

*Scatopsciara tricuspidata* (Winnertz, 1867)

= *degenerans* (Frey, 1948)

*Scatopsciara vitripennis* (Meigen, 1818)

**sg. *Xenopygina*** Frey, 1948

*Scatopsciara gabyae* (Heller, 1998)

*Scatopsciara obliqua* Vilkamaa, Hippa & Heller, 2013

*Scatopsciara paradoxa* (Frey, 1948)

*Scatopsciara simillima* (Tuomikoski, 1960)

***SCHWENCKFELDINA*** Frey, 1942

*Schwenckfeldina carbonaria* (Meigen, 1830)

*Schwenckfeldina pectinea* Menzel & Mohrig, 1991

***SCIARA*** Meigen, 1803

*Sciara flavimana* Zetterstedt, 1851

*Sciara hebes* (Loew, 1869)

= *mendax* Tuomikoski., 1960

= *nursei* Freeman, 1983

= *ulrichi* Menzel & Mohrig, 1998

*Sciara helvola* Winnertz, 1867

*Sciara hemerobioides* (Scopoli, 1763)

= *thomae* (Linnaeus, 1767)

*Sciara humeralis* Zetterstedt, 1851

*Sciara lackschewitzi* (Lengersdorf, 1934)

***SCYTHROPOCHROA*** Enderlein, 1911

*Scythropochroa quercicola* (Winnertz, 1869)

*Scythropochroa radialis* Lengersdorf, 1926

***TRICHOSIA*** Winnertz, 1867

= *Leptosciara* Frey, 1942

= *Lestremioides* Frey, 1942

***Trichosia*** Winnertz, 1867

*Trichosia acrotricha* Tuomikoski, 1960

*Trichosia borealis* (Frey, 1942)

*Trichosia confusa* Menzel & Mohrig, 1997

*Trichosia flavicoxa* Tuomikoski, 1960

*Trichosia diota* (Garrett, 1925)

*Trichosia glabra* (Meigen, 1830)

*Trichosia habilis* (Johannsen, 1912)

= *edwardsi* (Lengersdorf, 1930)

*Trichosia morio* (Fabricius, 1794)

= *caudata* (Walker, 1848)

*Trichosia splendens* Winnertz, 1867

*Trichosia ussurica* Mohrig & Antonova, 1978

***XYLOSCIARA*** Tuomikoski, 1957

**sg. *Protoxylosciara*** Tuomikoski, 1960

*Xylosciara longiforceps* (Bukowski & Lengersdorf, 1936)

**sg. *Xylosciara*** Tuomikoski, 1957

*Xylosciara heptacantha* Tuomikoski, 1957

*Xylosciara lignicola* (Winnertz, 1867)

*Xylosciara microdon* (Frey, 1948)

*Xylosciara misella* (Frey, 1948)

*Xylosciara phryganophila* (Frey, 1948)

*Xylosciara senta* Vilkamaa, Hippa & Heller, 2013

*Xylosciara spinata* (Pettey, 1918)

= *betulae* Tuomikoski, 1960

*Xylosciara steleocera* Tuomikoski, 1960

*Xylosciara trimera* Tuomikoski, 1960

*Xylosciara validinervis* Tuomikoski, 1960

***ZYGONEURA*** Meigen, 1830

**sg. *Allozygoneura*** Menzel & Mohrig, 1998

*Zygoneura calthae* Tuomikoski, 1960

**sg. *Zygoneura*** Meigen, 1830

*Zygoneura sciarina* Meigen, 1830

**UNPLACED IN SCIAROIDEA**

***SCIAROSOMA*** Chandler, 2002

*Sciarosoma nigriclava* (Strobl, 1898)

= *borealis* Chandler, 2002

## Excluded species

*Lycoriella auripila* (Winnertz, 1867), misidentified in Tuomikoski (1960).

*Bradysia betuleti* (Lengersdorf, 1940), misidentified in Tuomikoski (1960).

*Bradysia subbetuleti* Mohrig & Krivosheina, 1989, misidentified in Salmela and Vilkamaa (2005).

*Corynoptera concinna* (Winnertz, 1867), misidentified in Tuomikoski (1960).

*Corynoptera bistrispina* (Bukowski & Lengersdorf, 1936), misidentified in Tuomikoski (1960).

*Trichosia trochanterata* (Zetterstedt, 1851), misidentified in Tuomikoski (1960).
